# Letter to the Editor: Faecal Immunochemical Test (FIT) Sensitivity: A Five Year Audit

**DOI:** 10.3389/bjbs.2024.13381

**Published:** 2024-07-26

**Authors:** Waseem Jerjes

**Affiliations:** ^1^ Research and Development Unit, Hammersmith and Fulham Primary Care Network, London, United Kingdom; ^2^ Faculty of Medicine, Imperial College London, London, United Kingdom

**Keywords:** sensitivity, faecal immunochemical test (FIT), colorectal cancer (CRC), false negatives, primary care implications

## Introduction

The study by Cole et al. [1] tackles a very important issue on early detection of colorectal cancer (CRC) and reports on the diagnostic sensitivity of Faecal Immunochemical Test (FIT) in clinical practice. This study is important from my perspective as a general practitioner (GP), with relevance to general practice, patient outcomes and the entire healthcare system.

The important outcomes from this rigorous and methodological 5-year audit have given an insight into the real-life scenarios of FIT performance in regards to its sensitivity and the false negative rate. The sensitivity of 92.00%, as reported, of FIT is subjective; FIT, although a good screening tool, is also prone to failure at times. False negative appearances to occur in 8.00% of cases, so suspicion should remain high in symptomatic patients even with a negative FIT [1].

### Primary Care Implications

GPs play an important role in the early detection and management of CRC. As a primary care physician, when patients come to us with GI complaints, we tend to focus on using FIT to pre-try the diagnosis. Because a positive FIT is so sensitive, a FIT-positive is an URGENT 2ww referral [2, 3]. Nonetheless, this dichotomy was not present in the study by Cole et al. It raises an important controversy: false negatives, especially for flat or sessile lesions that do not bleed enough for detection by FIT [1].

This finding is of considerable significance for GPs. It makes a strong point of the value of a holistic clinical assessment. Regardless of FOBT results, if patients have symptoms such as changes in bowel habit, unexplained weight loss, iron deficiency anaemia then they should go onto further investigation. In high-risk symptoms patients, absence of FIT positivity, should not influence the decision to refer to further investigation, e.g., colonoscopy. This is consistent with the guidance of the Association for Coloproctology of Great Britain and Ireland recommending not to use the FIT test as the sole basis for excluding people from referral for suspected colorectal cancer [4].

In addition, the research highlights the importance of GPs recognising the constraints of FIT. Besides, patient assessment of how to collect samples is critical in avoiding pre-analytical errors that spoil any accuracy of the test [5]. More fundamentally, GPs would need to be aware of the potential for biological factors such as lesion morphology and the degradation of haemoglobin in the gastrointestinal tract to impact on FIT results [6].

The results suggest that patients may benefit from more explicit information on what their test is for and what it implies. It is important that patients know that while FIT is a useful test, it is not the gold standard. They should be encouraged to seek follow-up for persistent symptoms regardless of their negative FIT result. This knowledge is important in order to secure that follow-up is timely and adequate to avoid delay in CRC diagnosis and treatment.

The fact that the study identified false negatives in 36 patients, with 6 of those referring to surgically removed masses that histologically were sessile lesions, also underscores the importance of following up patients with recommendations. Patients with negative FIT results and continuing symptoms should be educated that further diagnostic testing is warranted. This could mean reinforcing the need to maintain a low level of suspicion for symptoms to repeat FIT or pursue other diagnostic tests such as colonoscopy.

### Secondary Care Implications

These data should prompt secondary care providers to carefully consider how to modify referral pathways and diagnosis protocols. Cole et al. data indicate that FIT may not be an adequate test for the diagnosis of CRC in symptomatic individuals alone. It is important for the secondary care providers and GPs to collaborate and aim to give suggested referrals to those with persistent symptoms for timely and thorough evaluations.

The study also suggests a potential reason for the creation of additional or otherwise improved colorectal cancer diagnostic resources to couple with FIT. This might include introducing other biomarkers, imaging methodologies, or molecular tests to create a more complete view of CRC risk. Integration of primary together with secondary care is important for development of a stepwise diagnostic pathway with minimal risk for omissions of diagnoses [7].

## Discussion

The broad implications of the results of the study, also regarding healthcare policy and resource allocation. This marked drop of CRC diagnoses during the COVID-19 pandemic, and a sharp reversal in 2021, may show the significant effects that healthcare access delays can bear on cancer detection rates. This reinforces the importance of strong health systems that are able to provide required services including cancer screening during a public health crisis.

Healthcare policymakers might wish to invest in public health campaigns to raise awareness around the importance of CRC screening and to the limitations of FIT. Such a campaign could help to get people showing symptoms to come forward as quickly as possible. Furthermore, it is essential to secure funding and resources for CRC screening programs to help to sustain high diagnostic performance and early detection of CRC.

The findings, the authors contend, highlight the need for continuous education and training for healthcare providers. Regular updates on the latest evidence concerning FIT and its limitations should especially be provided to GPs. It emphasises the essential need of an exhaustive clinical evaluation and stringent follow-up in patients presenting with alarming features even if the FIT is negative for ongoing symptoms.

Additionally, this study emphasises the potential benefit of interdisciplinary cooperation in CRC care. Collaborative efforts among GPs, gastroenterologists, oncologists, and other healthcare professionals are crucial for developing integrated care pathways that account for the limitations of FIT and aim for improved timely diagnosis and treatment of CRC.

Moreover, the characteristics of false-negative FIT results should be explored further. Characterizing the biological and technical sources of these discrepancies could improve the test or the means by which it is employed. Longitudinal follow-up studies on patient-related outcomes following a positive FIT followed by diagnostic procedures (or not) would be ideal to further optimise screening protocols.

## Data Availability

The original contributions presented in the study are included in the article/Supplementary Material, further inquiries can be directed to the corresponding author.
